# HMSC-Derived Exosome Inhibited Th2 Cell Differentiation via Regulating miR-146a-5p/SERPINB2 Pathway

**DOI:** 10.1155/2021/6696525

**Published:** 2021-05-14

**Authors:** Jing Zhou, Yi Lu, Wei Wu, Yunhai Feng

**Affiliations:** ^1^Department of Otorhinolaryngology, Head & Neck Surgery, Dahua Hospital, Shanghai 200237, China; ^2^Department of PharmaceuticsSchool of Pharmacy, Fudan University, Shanghai 201203, China

## Abstract

**Background:**

Allergic rhinitis (AR) is a global disease without specific treatment. Human mesenchymal stem cell- (HMSC-) derived exosomes (HMSC-exos) have been implicated for the amelioration of allergic inflammation by delivering miR-146a-5p in a mouse asthma model. However, the antiallergic activity and the underlying mechanism of HMSC-exos in AR remain unclear. The present study aimed to investigate the role of HMSC-exos in the pathogenesis of AR.

**Materials and Methods:**

Blood specimens were collected from AR patients and healthy donators for investigation. HMSC and CD4^+^ T cells were used in the present study. Flow cytometry was used to characterize the population of Type 1 helper T (Th1) and Th2 cells. Specific siRNA and overexpressed plasmids were designed to silence or overexpress the expressions of miR-146a-5p and SERPINB2. Luciferase reporter assay was adopted to explore the binding site of miR-146a-5p and SERPINB2. Quantitative real-time PCR and immunoblots were performed to estimate the expression of target genes.

**Results:**

The population of Th2 cells was significantly elevated in AR patients as compared with that in healthy donators. HMSC-exos could decrease the expression of SERPINB2 and the differentiation of Th2 cells. miR-146a-5p in HMSC-exos exhibited consistent effects and lowered the expression of SERPINB2 by binding on its 3′UTR region. Moreover, the differentiation of Th2 cells was promoted by SERPINB2 that could be reversed by HMSC-exos. Additionally, the miR-146a-5p expression was negatively associated with the SERPINB2 expression in the serum of AR patients.

**Conclusion:**

HMSC-exos could inhibit the differentiation of Th2 cells via the regulation of the miR-146a-5p/SERPINB2 pathway. miR-146a-5p and SERPINB2 could be applied as potential targets for AR treatment.

## 1. Introduction

Allergic rhinitis (AR) is a type of nasal mucosal disease characterized by overreacted immune responses. It is a global disease that affects approximately 20%–30% of the world population [1]. Although AR is not fatal, patients usually experience physical discomfort and psychological stress that heavily impairs their quality of life. The pathogenesis of AR is associated with the activation of immunoglobulin E (IgE) and the release of inflammatory components, such as histamine in response to specific allergens [2]. Our previous study revealed that the reduction of inflammatory responses using taurine could ameliorate the symptoms of AR in a mouse model [3]. Therefore, strategies to inhibit the inflammation in the nasal mucosa are potential therapeutics for AR treatment.

Type 1 helper T (Th1) and Th2 cells are two subtypes of helper T cells. Th1 cells are activated by IL-12 and marked by T-bet. Th1 cells can secrete IFN-*γ* and IL-2 and stimulate the activities of macrophages and CD8^+^ T cells that mediate cell-mediated immune responses against intracellular antigens [4]. By contrast, Th2 cells are activated by IL-4 and IL-2 and marked by GATA-3. Th2 cells can stimulate B cells and mediate humoral immune responses against extracellular antigens [4]. The imbalance of Th1 and Th2 cells is considered to be a pathogenic factor of allergic diseases, such as AR [5]. During AR, the CD4^+^ T cells are more likely to differentiate into Th2 cells that reduce the population of Th1 cells and result in the imbalance of Th1/Th2 cells. Moreover, the inhibition of Th2 cells was a potential approach for AR treatment [6]. Therefore, therapeutics targeting Th1 and Th2 cells are promising approaches for AR treatment.

Exosomes are believed to play a critical role in the orchestration of immune responses. Previous studies have indicated that cell-derived exosomes could trigger proinflammatory responses by the transportation of molecules contained in the exosomes [7, 8]. Additionally, the released exosomes and microRNAs (miRNAs) are detected in bronchoalveolar lavage fluids from patients with respiratory diseases, such as asthma and AR, indicating that exosomes and miRNAs are involved in AR pathogenesis [9]. A previous study showed that exosomes derived from the human mesenchymal stem cell (HMSC) could ameliorate inflammation by delivering miR-146a-5p that was abundantly expressed in HMSC-derived exosomes (HMSC-exos) [10].

Serpin family B member 2 (SERPINB2), a member of the serine protease inhibitor family, is predicted as a potential target of miR-146a-5p that can stimulate inflammation. Moreover, SERPINB2 was highly expressed in nasal brushings of AR patients and played a crucial role in Th2-mediated immune responses [11]. By searching the predicting miRNA for SERPINB2 using the TargetScan database (http://www.targetscan.org), SERPINB2 is predicted as a potential target of miR-146a-5p. However, the regulatory mechanisms of miR-146a-5p and SERPINB2 in AR remain unclear. Furthermore, their role in the differentiation of Th1 and Th2 cells remains unclear. Therefore, our study was conducted to explore the role of the miR-146a-5p/SERPINB2 signaling pathway in the differentiation of Th1 and Th2 cells, aiming to provide novel strategies for AR treatment.

## 2. Materials and Methods

### 2.1. Sample Collection

The blood specimens of AR patients and healthy donators were collected from Dahua Hospital, Shanghai, China, after written informed consent was obtained from all the subjects (*n* = 25 for each group). All blood samples were subpacked in 500 *μ*L sodium citrate (3.2%) tubes, centrifuged, and the plasma snap-frozen in liquid N_2_ and stored at -80°C for further analysis. These AR patients, aged 20–50years, do not have any other chronic medical conditions or allergic disorders except AR. The inclusion and exclusion criteria were as per those mentioned in a previous report [12]. Briefly, the inclusion criteria were as follows: age 20–50years, daytime fatigue, daytime somnolence, nasal congestion, perennial AR with a positive skin test response for perennial allergen (wheal diameter equal to 3 mm or greater), and a negative skin test response for seasonal allergens. The exclusion criteria included seasonal allergies, known sleep apnea, obesity, nasal polyps, recent upper respiratory tract infection, deviated septum, asthma, and other respiratory diseases. Age-matched healthy individuals without chronic medical conditions or allergic disorders were selected as controls.

### 2.2. Cell Culture

The HMSC and 293T cells were obtained from Shanghai Biology Institute (Shanghai, China). HMSC was obtained by density centrifugation isolation from bone marrow, then cultured and expanded. Cells were cultured in DMEM medium with 10% fetal bovine serum (FBS, Gibco, USA) and were maintained in 5% CO_2_ at 37°C. Flow cytometry was used to detect biomarkers of HMSC, including positive biomarkers (CD90 and CD105) and negative biomarkers (CD34 and CD45).

### 2.3. Exosomes Isolation

Exosomes were precipitated using exosome precipitation solution (Exo-Quick; System Bioscience) as per the manufacturer's instructions with some modifications. HMSC exosomes were collected from approximately 3.2 × 10^7^ cells at early passages (passages 2–3). Once the HMSC cultures reached 70% confluence, the cells were cultured for 24–48 h in *α*-MEM containing exosome-depleted FBS or PL. Exosome-depleted FBS and PL were obtained with overnight centrifugation at 70,000 × g at 4°C. Briefly, the HMSC conditioned medium was centrifuged twice at 500 × g for 10 min, twice at 2000 × g for 15 min, and twice at 10,000 × g for 30 min. The supernatant was then transferred to Ultra-Clear tubes and centrifuged at 70,000 × g for 1 h at 4°C in an SW32Ti rotor (Beckman Coulter Inc., Woerden, The Netherlands). The exosome-containing pellet was washed with PBS and centrifuged at 70,000 × g for 1 h. The pellet was then carefully suspended in 200 *μ*L PBS and used immediately or stored at −80°C. Exosome protein markers, CD63 (Ab134045, Abcam, UK), CD81 (ab109201, Abcam, UK), and TSG101 (ab125011, Abcam, UK), were determined using immune blotting. The morphology of the exosomes was examined using transmission electron microscopy. The size analysis of the exosome was provided in supplementary material file 1.

### 2.4. Exosome Uptake Assay

Exosomes were stained with PKH67 (Sigma) as per previously reported protocols [13]. The exosomes were incubated with PKH67 solution for 4 min at room temperature. Then, exosomes were isolated via centrifugation at 100,000 × g for 1 h. When the exosomes were cocultured with CD4^+^ T cells, the uptake of exosomes was detected using confocal microscopy.

### 2.5. Isolation of CD4^+^ T Cells

In the present study, the blood samples from AR patients or normal corresponding donators were diluted with PBS solution (1 : 1). Peripheral blood mononuclear cells were obtained via centrifugation on a lymphocyte separation medium. Then, the cell concentration of lymphocytes was adjusted to 1 × 106/mL. The human CD4^+^ T cells were isolated with CD4^+^ T cell isolation kits (130-096-533, Miltenyi Biotec, Germany). All the procedures were performed as per the manufacturer's instructions.

### 2.6. RNA Isolation and qRT-PCR Analysis

Total RNA of samples was extracted using TRIzol (Invitrogen) as per the manufacturer's protocol from different CD4^+^ T cells from AR patients. RNA was transcribed into cDNA using a cDNA synthesis kit (RR047A, Takara). Quantitative real-time PCR was performed using SYBR green (RR820A, Takara) as per the three-step amplification procedure. Gene expression was calculated using the 2^−*ΔΔ*Ct^ method. All the data represent the average of three replicates. The detection of genes was performed using the following primers: hsa-miR-146a-5p, F: CGCGTGAG AACTGAATTCCA, R: AGTGCAGGGTCCGAGGTATT; SERPINB2, F: CGAGGAGAGGAGAT TGAAAC, R: GGATCTGCTG CATGAAC-; T-bet, F: TTGAGGTGAACGACGGAGAG, R: TGGGTAGGAGAGGAGAGTAGTG; GATA-3, F: GAGCGAGCAACGCAATCTGAC, R: AGGCTGGGAAGCAAAGGTGAG; *β*-actin, F: GATGACCCAGATCATGTTTGAG, R: TAATGTCACGCACGATTTCC′; U6, F: CTCGCTTCGGCAGCACA, R: AACGCTTCACGAATTTGCGT.

### 2.7. Western Blot Analyses

Different CD4^+^ T cells, as indicated above, were lysed using RIPA lysis buffer with a protease inhibitor to obtain total proteins (Beyotime, China). The protein was separated in SDS-PAGE and transferred to a polyvinylidene fluoride membrane. Thereafter, the primary antibody was applied at 4°C overnight. The secondary antibody (Beyotime, Shanghai, China) was applied for 1 h at 37°C. The protein expression was detected after the application of ECL substrate. All the data represent the average of three replicates. Our study used the following primary antibodies: TSG101 (Ab125011, Abcam), CD63 (Ab59479, Abcam), CD81 (Ab109201, Abcam), SERPINB2 (Ab269275, Abcam), T-bet (Ab91109, Abcam), GATA-3 (Ab106625, Abcam), and *β*-actin (20536-1-AP, Proteintech).

### 2.8. Flow Cytometry

The population of Th1 and Th2 cells was estimated using Th1/Th2/Th17 PhenotypingKit (560758, BD Biosciences). Total 1 × 10^5^ CD4^+^ T cells were collected and stained with PerCP-Cy5.5-labeled anti-IFN*γ* and APC-anti-IL-4 for 30 min at room temperature. Th1 cells were marked as CD4^+^IFN-*γ*^+^, whereasTh2 cells were marked as CD4^+^IL-4^+^. The populations of Th1 and Th2 cells were calculated using the FACSDiva 7.0 software. All the data represent the average of three replicates.

### 2.9. Knockdown and Overexpression of SERPINB2

Cells were transfected with three siRNAs targeting at SERPINB2 (RiboBio, Guangzhou, China) and a plasmid containing the coding sequence of human SERPINB2 (Major, Shanghai, China). Two negative control (NC) plasmids were applied as the control group of the siRNA and overexpression plasmid. Lipofectamine 3000 (Thermo Fisher Scientific) was used for the transfection. The sequence of SERPINB2 plasmids was provided as follows: siSERPINB2-1: 5′-CCTTATACAAGTTACTTAA-3′; siSERPINB2-2: 5′-GGCACAAGCTGCAGATAAA-3′; siSERPINB2-3: 5′-GGTCAAGACTCAAACCAAA-3′; siNC: 5′-CAGUACUUUUGUGUAGUACAA-3′.

### 2.10. Dual-Luciferase Reporter Gene Assay

Human embryonic kidney cells (HEK 293T) are widely used for determining the dual-luciferase reporter gene assay [14]. Wildtype and mutant sequences of SERPINB2 were cloned to luciferase reporter vectors (pGL3-Basic). Then, 293T cells were transfected with vectors and miR-146a-5p inhibitor or mimics. After 48 h, a dual-luciferase reporter gene kit (Beijing Yuanpinghao Biotechnology Co., Ltd.) was used to detect and analyze the luciferase activity. All the data represent the average of three replicates.

### 2.11. Statistical Analyses

Statistical analyses were conducted using GraphPad Prism version 7.0. The experimental data are presented as mean ± SD for at least three samples. Comparison between two groups was performed using the *T*-test, whereas comparison among multiple groups was performed with one-way analysis of variance. A two-sided *p* value of <0.05 was considered to indicate statistical significance.

## 3. Results

### 3.1. The Population of Th2 Cells Was Elevated in the CD4^+^ T Cells of AR Patients

**Figure 1:**
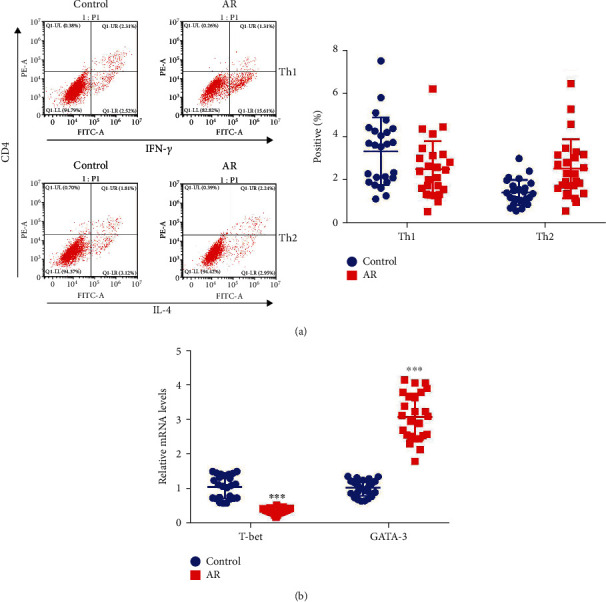
The population of Th2 cells was upregulated in the blood samples of allergic rhinitis (AR) patients. (a) Flow cytometry was used to examine the distribution of Th1 (CD4^+^IFN-*γ*^+^) or Th2 (CD4^+^IL-4^+^) cells in the blood samples of allergic rhinitis patients and corresponding donors. ^∗^*p* < 0.05 vs. control, ^∗∗^*p* < 0.01 vs. control. (b) qRT-PCR was used to examine the relative mRNA levels of T-bet (Th1 marker) and GATA-3 (Th2 marker) in the blood-derived mononuclear cells of allergic rhinitis patients and corresponding donors. ^∗∗∗^*p* < 0.001 vs. control.

To verify the ratio of Th1 and Th2 cells in AR, we collected blood specimens from AR patients for analyses. The CD4^+^ T cells were isolated from the blood specimens. Flow cytometry showed that the quantity of Th1 (CD4^+^IFN-*γ*^+^) was significantly decreased, whereas that of Th2 (CD4^+^IL-4^+^) was significantly increased in AR patients as compared with that in healthy donators (*p* < 0.05) (Figure 1(a)). Moreover, the expression of T-bet (the marker of Th1 cells) was significantly decreased, whereas that of GATA-3 (the marker of Th2 cells) was significantly elevated in AR patients as compared with that in healthy donators (*p* < 0.05) (Figure 1(b)). These results indicated that the elevation of Th2 might be associated with the development of AR.

### 3.2. Exosomes of HMSC Could Be Absorbed by CD4^+^ T Cells through Endocytosis in AR

**Figure 2:**
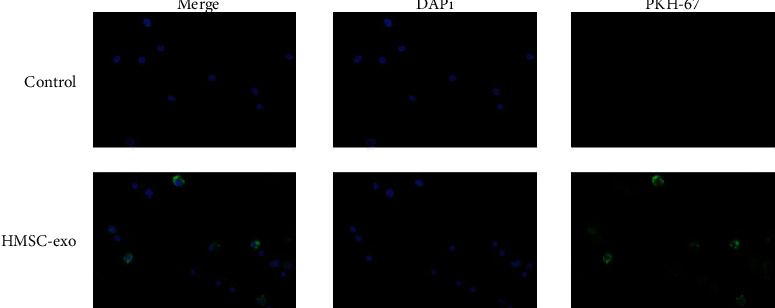
The HMSC-exo was able to uptake by AR CD4^+^ cells through endocytosis. The PKH67-labeled (green) immature HMSC-exos were cocultured with CD4^+^ cells. Then, the AR CD4^+^ cells were fixed and stained with DAPI (blue). The uptake of HMSC-exos by AR CD4^+^ cells was observed under a confocal microscope.

Furthermore, we explored the interaction between HMSC-exos and CD4^+^ T cells. The HMSC-exos were isolated, as described previously. We verified the isolated HMSC using flow cytometry that revealed that the positive rate of negative biomarkers (CD34 and CD45) for HMSC was <1.0%, whereas that of positive biomarkers (CD90 and CD105) for HMSC was >90% (Figure S1A). Transmission electron microscopy validated the morphology of the isolated HMSC-exos (Figure S1B). Then, the markers of exosomes, including TSG101, CD63, and CD81, were detected in isolated exosomes that indicated the successful isolation of HMSC-exos (Figure S1C). When CD4^+^ T cells were cocultured with PKG67-labeled HMSC-exos, we found that CD4^+^ T cells could absorb HMSC-exos via endocytosis (Figure 2). These results suggested that HMSC could interact with CD4^+^ T cells via the secretion of exosomes.

### 3.3. HMSC-Exos Decreased the Expression of SERPINB2 and Differentiation of Th2 Cells after Coculture with CD4^+^ T Cells

**Figure 3:**
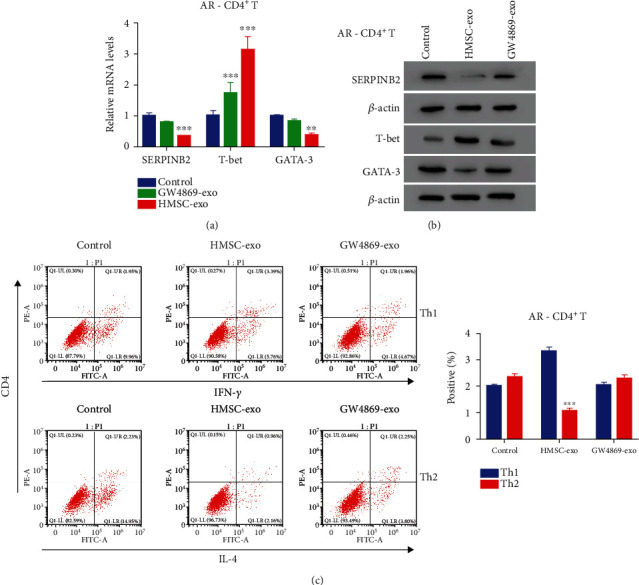
The differentiation of AR CD4^+^ cells into Th2 type was downregulated after co-culture with HMSC-exo. (a, b) qRT-PCR and western blot analysis were used to examine the relative mRNA and protein levels of SERPINB2, T-bet, and GATA-3 in AR CD4^+^ after coculture with GW4869-exo or HMSC-exo. ^∗∗^*p* < 0.01 vs. control, ^∗∗∗^*p* < 0.001 vs. control. (c) The AR CD4^+^ cells differentiated into Th2 type cells were deeply suppressed after co-cultured with HMSC-exo. ^∗∗∗^*p* < 0.001 vs. Th1.

To understand the effects of HMSC-exos on CD4^+^ T cells, we detected the expression of several genes, including T-bet and GATA-3 of CD4^+^ T cells after coculture with HMSC-exos. GW4869 was added to inhibit the secretion of exosomes as an NC. The expression of T-bet was significantly elevated after coculture withGW4869-exos and HMSC-exos, whereas that of GATA-3 was significantly decreased after coculture with HMSC-exos (*p* < 0.05) (Figures 3(a) and 3(b)). In the meantime, the SERPINB2 expression was significantly reduced after coculture with HMSC-exos, consistent with that in GATA-3 (*p* < 0.05). Moreover, flow cytometry revealed that the population of Th2 cells was significantly decreased after coculture with HMSC-exos (*p* < 0.05) (Figure 3(c)). However, the population of Th2 cells was not significantly altered when they were cocultured with GW4869-exos (*p* < 0.05). These results indicated that HMSC-exos could reduce the expression of SERPINB2and differentiation of Th2 cells.

### 3.4. HMSC-Exos Inhibited the Differentiation of Th2 Cells via Delivery of miR-146a-5p

**Figure 4:**
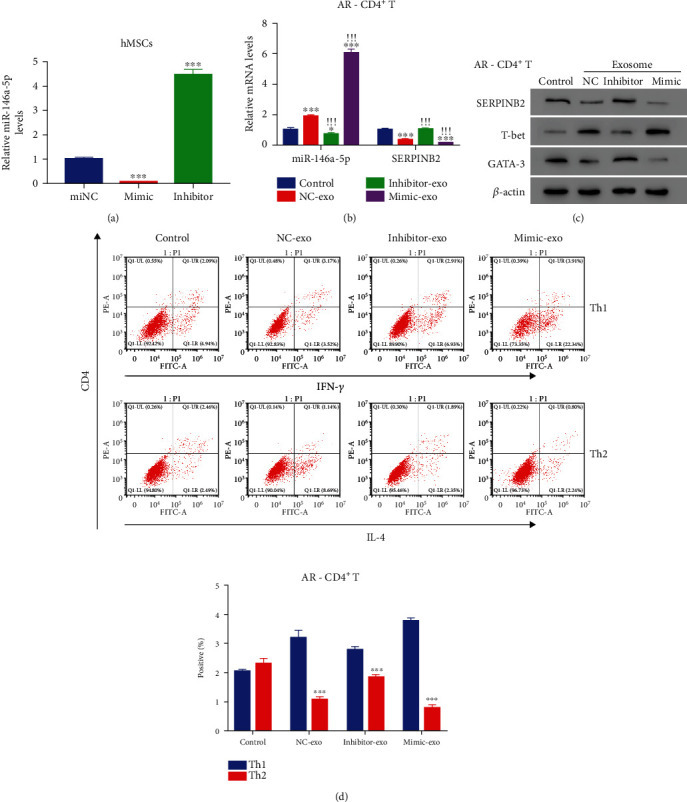
HMSC-derived exosome inhibited the AR CD4^+^ cells differentiated into Th2 type cells through delivering miR-146a-5p. (a) miR-146a-5p induced silencing and overexpression using corresponding mimic and inhibitor in hMSCs. ^∗∗∗^*p* < 0.001 vs. miNC (b). The relative levels of miR-146a-5p and SERPINB2 in AR CD4^+^ cells after coculture with NC-exo, inhibitor-exo, and mimic-exo. ^∗^*p* < 0.05 vs. control, ^∗∗∗^*p* < 0.001 vs. control; !!! ^∗^*p* < 0.05 vs. NC-exo. (c) Western blot analysis was used to quantify the protein contents of SERPINB2, T-bet, and GATA-3 in AR CD4^+^ cells after co-culture with NC-exo, inhibitor-exo, and mimic-exo. (d) The distribution of Th1 and Th2 cells in AR CD4^+^ T cells after co-culture with NC-exo, inhibitor-exo, and mimic-exo. ^∗∗∗^*p* < 0.001 vs. control.

Furthermore, we explored the underlying mechanism of HMSC-exos in the differentiation of Th1 and Th2 cells. The mimics and inhibitor of miR-146a-5p were used to induce the upregulation and downregulation of miR-146a-5p in HMSC (*p* < 0.05) (Figure 4(a)). miR-146a-5p mimics exosomes (mimic-exo) significantly elevated, whereas miR-146a-5p inhibitor exosomes (inhibitor-exo) significantly decreased the expression of miR-146a-5pas compared with the control group (*p* < 0.05) (Figure 4(b)). Moreover, SERPINB2 was significantly decreased by miR-146a-5p mimic-exo, whereas inhibitor-exo significantly promoted the SERPINB2 expression (*p* < 0.05). Furthermore, we confirmed the alteration of SERPINB2 induced by mimic-exo using immunoblots (Figure 4(c)). The expression of T-bet was markedly decreased after coculture with inhibitor-exo; the expression of GATA-3 was notably decreased after coculture with mimic-exo. Additionally, flow cytometry showed that the population of Th1 cells was significantly reduced by inhibitor-exo, whereas that of Th2 cells was significantly reduced by mimic-exo. These results indicated that HMSC-exos could inhibit the differentiation of Th2 cells by delivering miR-146a-5p.

### 3.5. miR-146a-5p Inhibited SERPINB2 by Binding on Its 3′UTR Region

**Figure 5:**
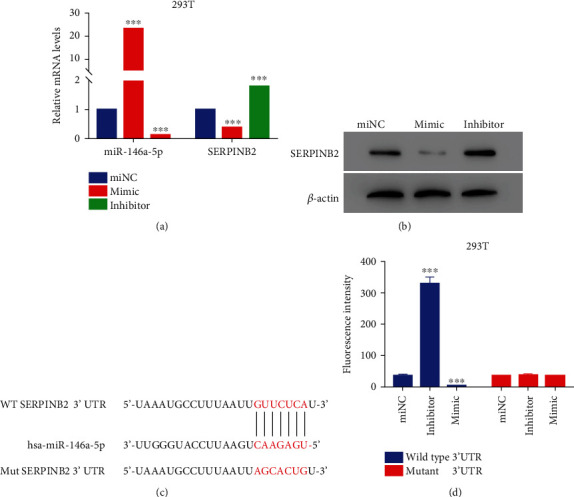
miR-146a-5p inhibited the transcription of SERPINB2 by binding on its 3′UTR. (a) qRT-PCR was used to examine the relative levels of miR-146a-5p and SERPINB2 in 293T cells after coculture with miR-146a-5p miNC, mimic, and inhibitor. ^∗∗∗^*p* < 0.001 vs. miNC. (b) The protein level of SERPINB2 was examined in 293T cells after coculture with miR-146a-5p miNC, mimic, and inhibitor. (c) The wild type and mutant binding site in the 3′UTR of SEEPINB2 for miR-146a-5p. (d) Dual-luciferase report vector was used to determine the relationship between miR-146a-5p and SERPINB2 in293T cells. ^∗∗∗^*p* < 0.001 vs. miNC.

SERPINB2 was significantly reduced with the elevated level of miR-146a-5p; therefore, we explored the correlation between miR-146a-5p and SERPINB2. The mimics and inhibitor of miR-146a-5p were used to induce the upregulation and downregulation of miR-146a-5p (*p* < 0.05) (Figure 5(a)). The expression of SERPINB2 was significantly elevated with miR-146a-5p inhibitor that was reversed by miR-146a-5p mimics (*p* < 0.05) (Figures 5(a) and 5(b)). The binding region of SERPINB2 and miR-146a-5p was predicted using online databases (Figure 5(c)). Dual-luciferase report vectors with wildtype and mutant sequences of 3′UTR of SERPINB2 were transfected in 293T cells. Results showed that the fluorescence intensity of the wildtype vector was significantly affected (*p* < 0.05), whereas the mutant vector exhibited no significant change (*p* > 0.05), indicating that miR-146a-5p could directly bind to the 3′UTR region of SERPINB2 (Figure 5(d)). These results suggested that SERPINB2 was involved in the biological activities of miR-146a-5p.

### 3.6. SERPINB2 Promoted the Differentiation of Th2 Cells and Was Suppressed by HMSC-Exos

**Figure 6:**
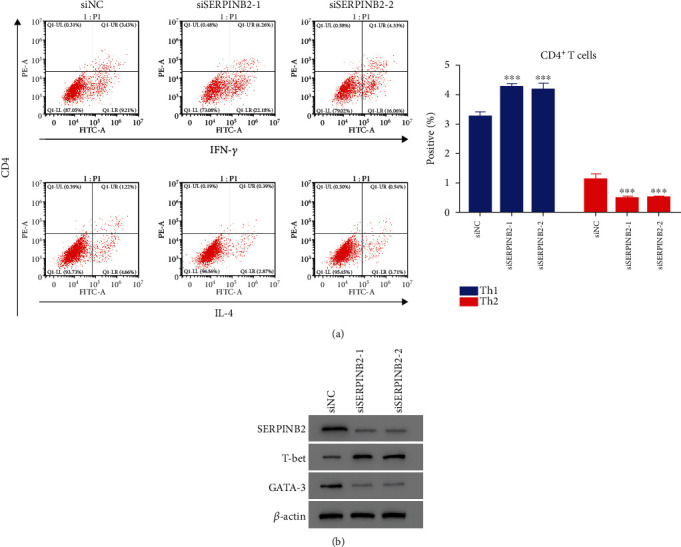
Knockdown of SERPINB2 inhibited the CD4^+^ T cells differentiated into Th2 type cells. (a) The differentiation of CD4^+^ T cells into Th2 type cells was deeply suppressed after transfection with siSERPINB2-1 or siSERPINB2-2. ^∗∗∗^*p* < 0.001 vs. siNC. (b) Western bot analysis was used to examine the protein levels of SERPINB2, T-bet, and GATA-3 in the CD4^+^ T cells after transfection with siSERPINB2-1 or siSERPINB2-2.

**Figure 7:**
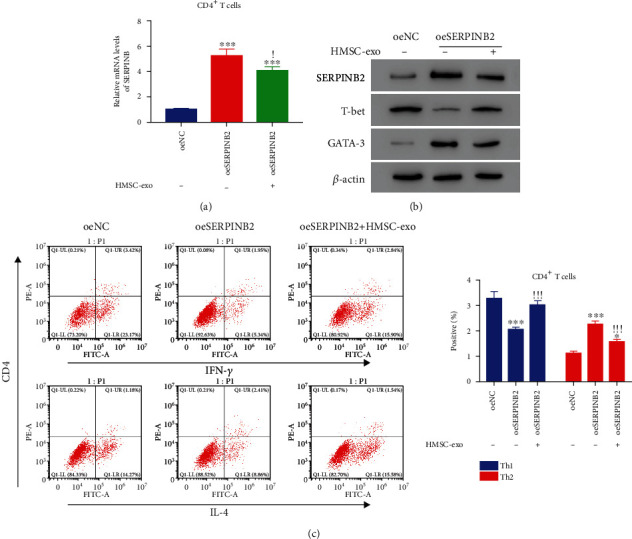
HMSC-derived exosome suppressed the effects of oeSERPINB2 in normal CD4^+^ T cells. (a) The level of SERPINB2 was downregulated in oeSERPINB2 cells after coculture with HMSC-exo. (b) Western blot analysis was used to examine the protein contents of SERPINB2, T-bet, and GATA-3 in oeSERPINB2 cells with or without coculture with HMSC-exo. (c) The differentiation of CD4^+^ T cells into Th2 cells was suppressed after coculture with HMSC-exo. ^∗^*p* < 0.05 vs. oeNC, ^∗∗∗^*p* < 0.001 vs. oeNC; !!! *p* < 0.001 vs. oeSERPINB2.

Furthermore, we explored the role of SERPINB2 in the differentiation of Th2 cells. Specific siRNAs and overexpression plasmids were designed to inhibit and elevate the SERPINB2 expression that was significantly inhibited by three siRNAs (*p* < 0.05) (Figure S2A–B). Moreover, SERPINB2 knockdown could significantly elevate the population of Th1 cells and diminish Th2 cells (*p* < 0.05) (Figure 6(a)). Moreover, T-bet was notably increased, and GATA-3 was decreased after SERPINB2 inhibition (Figure 6(b)). Additionally, the SERPINB2 expression was significantly increased after transfecting with SERPINB2-overexpressed plasmid (*p* < 0.05) (Figure S2C–D). HMSC-exos significantly reduced the SERPINB2 expression (*p* < 0.05) (Figure 7(a)). Additionally, the HMSC-exos markedly reduced the GATA-3 expression and increased the T-bet expression in CD4^+^ T cells (*p* < 0.05) (Figure 7(b)). Moreover, the population of Th1 cells did not change significantly, whereas that of Th2 cells decreased significantly after coculture with HMSC-exos (*p* < 0.05) (Figure 7(c)). These results indicated that SERPINB2 could promote the differentiation of Th2 cells, which was suppressed by HMSC-exos.

### 3.7. miR-146a-5p Was Negatively Associated with SERPINB2

**Figure 8:**
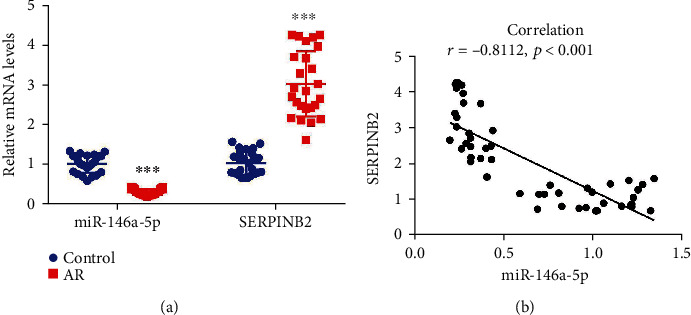
miR-146a-5p was negatively correlated with SERPINB2. (a) The relative levels of miR-146a-5p and SERPINB2 were examined in the serum-derived mononuclear cells of allergic rhinitis patients and corresponding donors, *n* = 25 for each group. (b) Correlation analysis between miR-146a-5p and SERPINB2.

To validate the association between miR-146a-5p and SERPINB2 in AR, we detected their expressions in AR patients and the corresponding donators. miR-146a-5p was significantly suppressed, whereas SERPINB2 was highly expressed in AR patients (*p* < 0.05) (Figure 8(a)). Moreover, miR-146a-5p was negatively associated with the SERPINB2 expression (*r* = −0.81, *p* < 0.05) (Figure 8(b)). These results suggested that miR-146a-5p and SERPINB2 were involved in the same signaling pathway.

## 4. Discussion

Our study investigated a novel miR-146a-5p/SERPINB2 signaling pathway in AR pathogenesis. The imbalance of Th1/Th2 cells was associated with AR pathogenesis. Exosomes derived from HMSC could inhibit the differentiation of Th2 cells via the delivery of miR-146a-5p. Moreover, SERPINB2 could promote the differentiation of Th2 cells that was suppressed by miR-146a-5p in HMSC-exos. These findings revealed a novel regulatory mechanism of HMSC-exos in the differentiation of CD4^+^ T cells and provided potential therapeutic targets for AR treatment.

AR is an allergic disease with overreacted immune responses in the nasal mucosa, wherein the generation of IgE and the recruitment of immune cells are involved in allergic responses [15]. The excessive activation of Th2 cells and the induction of eosinophilic-dependent inflammation are associated with AR symptoms [16]. Th2 cells are capable of releasing cytokines, including IL-4, IL-10, and IL-13, to stimulate the activities of different immune cells. IL-4 can stimulate the production of IgE antibodies by B cells that subsequently activates mast cells to produce histamine [17]. A previous study showed that these cytokines were elevated in the serum of AR patients [18]. In our study, the population of Th2 cells was significantly elevated in AR patients as compared with that in healthy donors. Thus, the elevation of Th2 cells was associated with AR pathogenesis.

Exosomes are crucial regulators of intercellular communications. The contents of the exosomes are believed to play key roles in allergic diseases. Exosomes derived from bronchial epithelial cells can stimulate chemotaxis and monocyte proliferation [19]. Additionally, macrophages can release exosomes to stimulate the inflammatory responses in response to intracellular pathogens [20]. Mast cells are critical for Th2- and IgE-mediated immune responses and can secrete histamine to stimulate inflammation [21]. A previous study has demonstrated that exosomes derived from mast cells could stimulate the secretion of inflammatory cytokines and induce airway inflammation and allergic symptoms [22]. Our study showed that HMSC-exos could be absorbed by CD4^+^ T cells and diminishes the Th2 cell population, indicating that HMSC-exos might be associated with the differentiation of CD4^+^ T cells during AR pathogenesis.

miR-146a-5p was first reported by Taganovet et al. in 2006 and was associated with the NF-*κ*B pathway [23]. TRAF6 and IRAK1 were predicted as potential targets of miR-146a-5p, and their expressions were suppressed by miR-146a-5p. Moreover, miR-146a-5p in the HMSC-exos could significantly inhibit the infiltration of immune cells, reduce the levels of Th2-related cytokines, and alleviate overreacted responses of the airway in a mouse asthma model [10]. Consequently, miR-146a-5p was considered to negatively regulate the immune responses. In our study, miR-146a-5p in the HMSC-exos was demonstrated to decrease the population of Th2 cells, consistent with previous hypotheses. Moreover, SERPINB2 was predicted as a potential target of miR-146a-5p. SERPINB2 is highly expressed during inflammation, infection, and tissue damage, indicating its immune features [24, 25]. A previous study revealed that SERPINB2 and miR-146a-5p are highly expressed in psoriatic skin and SERPINB2 was suppressed by miR-146a-5p [26]. Our study revealed that SERPINB2 inhibition could reduce the differentiation of Th2 cells, whereas its overexpression exhibited reversed effects. Moreover, the regulation by SERPINB2 could be reversed after coculture with HMSC-exos. These results indicated that HMSC-exos could inhibit Th2 cell differentiation via the miR-146a-5p/SERPINB2 pathway.

There are certain limitations of this study. Our results were mainly obtained from in vitro experiments. The construction of an AR mouse model will further help us investigate the biological activities of HMSC-exos in AR. However, our study still provides a novel molecular mechanism for AR pathogenesis.

## 5. Conclusions

In sum, our study showed that HMSC-exos could inhibit the differentiation of Th2 cells via the regulation of the miR-146a-5p/SERPINB2 pathway. miR-146a-5p and SERPINB2 could be applied as potential targets for AR treatment.

## Data Availability

The datasets used and/or analyzed during the current study are available from the corresponding author on reasonable request.

## Supplementary Materials

Supplementary Materials Supplementary materials: identification of HMSC-derived exosome. Figure S1: identification of human bone marrow mesenchymal stem cells (HMSC) and exosome. (a) Flow cytometer was used to examine the positive (CD90 and CD105) and negative biomarker (CD34 and CD45) for HMSC. (b) The morphology of exosome derived from HMSC. (c) Western blot was used to examine the protein levels of biomarker of exosome, including TSG101, CD63, and CD81. Figure S2: knockdown and overexpression of SERP1NB2 in normal CD4^+^ T cells. A and B. The relative mRNA and protein levels of SERP1NB2 were deeply suppressed in CD4^+^ T cells after transfecting with siSERP1NB2-1, siSERP1NB2-2, and siSERP1NB2-3. ^∗∗∗^*p* < 0.001 vs. siNC. (c, d) The relative mRNA and protein levels of SERP1NB2 were significantly overexpressed in CD4^+^ T cells after transfecting with oeSERP1NB2. ^∗∗∗^*p* < 0.001 vs. oeNC.
