# Supplementary material for: HMSC-Derived Exosome Inhibited Th2 Cell Differentiation via Regulating miR-146a-5p/SERPINB2 Pathway
Source: J Immunol Res. 2021 May 14;2021:6696525. doi: 10.1155/2021/6696525 (PMC8140841; doi:10.1155/2021/6696525)
Supplement: Supplementary Materials — identification of HMSC-derived exosome. Figure S1: identification of human bone marrow mesenchymal stem cells (HMSC) and exosome. (a) Flow cytometer was used to examine the positive (CD90 and CD105) and negative biomarker (CD34 and CD45) for HMSC. (b) The morphology of exosome derived from HMSC. (c) Western blot was used to examine the protein levels of biomarker of exosome, including TSG101, CD63, and CD81. Figure S2: knockdown and overexpression of SERP1NB2 in normal CD4+ T cells. A and B. The relative mRNA and protein levels of SERP1NB2 were deeply suppressed in CD4+ T cells after transfecting with siSERP1NB2-1, siSERP1NB2-2, and siSERP1NB2-3. ∗∗∗p < 0.001 vs. siNC. (c, d) The relative mRNA and protein levels of SERP1NB2 were significantly overexpressed in CD4+ T cells after transfecting with oeSERP1NB2. ∗∗∗p < 0.001 vs. oeNC. [file 6696525.f1.zip › Supplementart material file 1.pdf]

Electrophoresis & Brownian Motion
Video Analysis
Laser Scattering Microscopy

Operator (Report): ZetaView
Video Operator: ZetaView

Sample Parameters
Sample Name: UY0109_2
Comment: ZP PS100nm, Sample Remarks0:
Sample Remarks1:
Sample Remarks2:
Electrolyte: BI PBS
Temperature: 26.99 °C sensed
pH 7.0 entered
Conductivity: 15000.00 µS/cm sensed

Result (sizes in nm)

	Number	Concentration	Volume
Median (X50)	99.7	99.7	135.3
Span	36.4	36.4	58.7

Concentration: 4.9E+7 Particles / mL
Dilution Factor: 50
Original Concentration: 2.4E+9 Particles / mL

Quality
Average Counted Particles per Frame: 141
Number of Traced Particles: 1368

Measurement Parameters
Cell S/N: CA16-122-0096

Measurement Mode: Size Distribution 2 Cycles
11 Positions, 3 Removed for Analysis

Analysis Parameters
Max Area: 1000, Min Area: 10, Min Brightness: 30

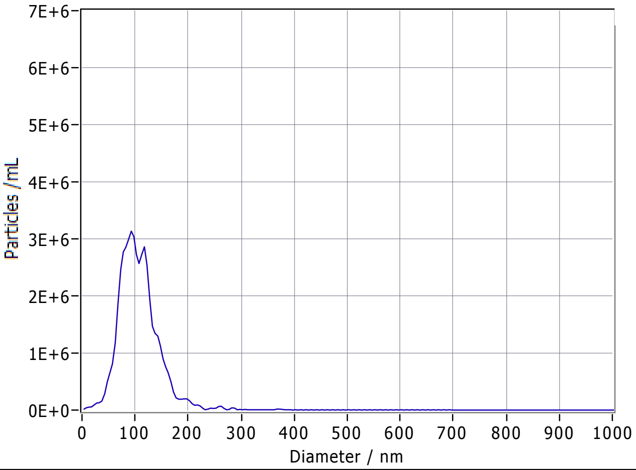

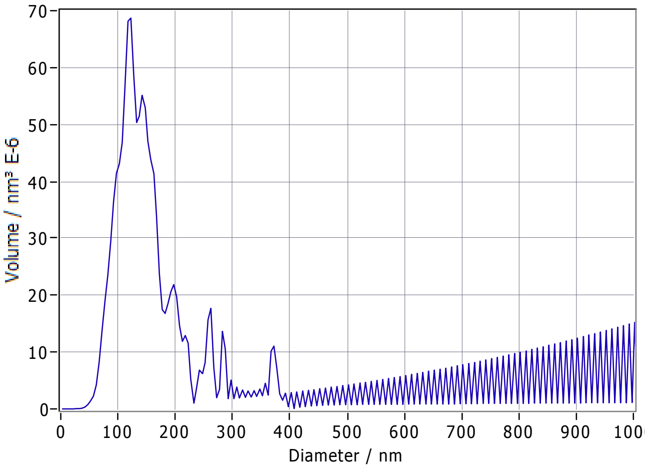

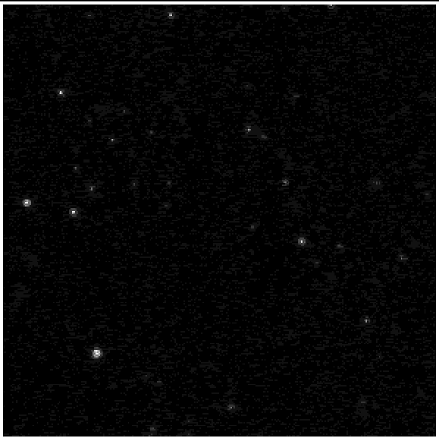

Peak Analysis (Concentration)

Diameter / nm	Particles/mL	FWHM / nm	Percentage
92.7	3.1E+6	65.9	98.4
260.6	6.5E+4	13.4	0.6
283.2	4.0E+4	9.5	0.2
375.6	1.1E+4	13.6	0.1
327.3	6.0E+3	5.5	0.1

X Values

	Number	Concentration	Volume
X10	64.6	64.6	90.0
X50	99.7	99.7	135.3
X90	148.4	148.4	221.4
Span	0.8	0.8	1.0
Mean	106.5	106.5	150.9
StdDev	36.4	36.4	58.7

Comment

(Signature)

Analyzed Video: Z:\ZetaViewResults\20200331\UMIBIO-8\20200331_0027_UY0109_2_size.avi

ZetaVIEW S/N 252, Software ZetaView 8.04.02 SP2, Camera 0.703 µm/px

Experiment: 2020-03-31 15:13, Report: 2020-03-31 15:17
