# Supplementary material for: HMSC-Derived Exosome Inhibited Th2 Cell Differentiation via Regulating miR-146a-5p/SERPINB2 Pathway
Source: J Immunol Res. 2021 May 14;2021:6696525. doi: 10.1155/2021/6696525 (PMC8140841; doi:10.1155/2021/6696525)
Supplement: Supplementary Materials — identification of HMSC-derived exosome. Figure S1: identification of human bone marrow mesenchymal stem cells (HMSC) and exosome. (a) Flow cytometer was used to examine the positive (CD90 and CD105) and negative biomarker (CD34 and CD45) for HMSC. (b) The morphology of exosome derived from HMSC. (c) Western blot was used to examine the protein levels of biomarker of exosome, including TSG101, CD63, and CD81. Figure S2: knockdown and overexpression of SERP1NB2 in normal CD4+ T cells. A and B. The relative mRNA and protein levels of SERP1NB2 were deeply suppressed in CD4+ T cells after transfecting with siSERP1NB2-1, siSERP1NB2-2, and siSERP1NB2-3. ∗∗∗p < 0.001 vs. siNC. (c, d) The relative mRNA and protein levels of SERP1NB2 were significantly overexpressed in CD4+ T cells after transfecting with oeSERP1NB2. ∗∗∗p < 0.001 vs. oeNC. [file 6696525.f1.zip › supplementary file.docx]

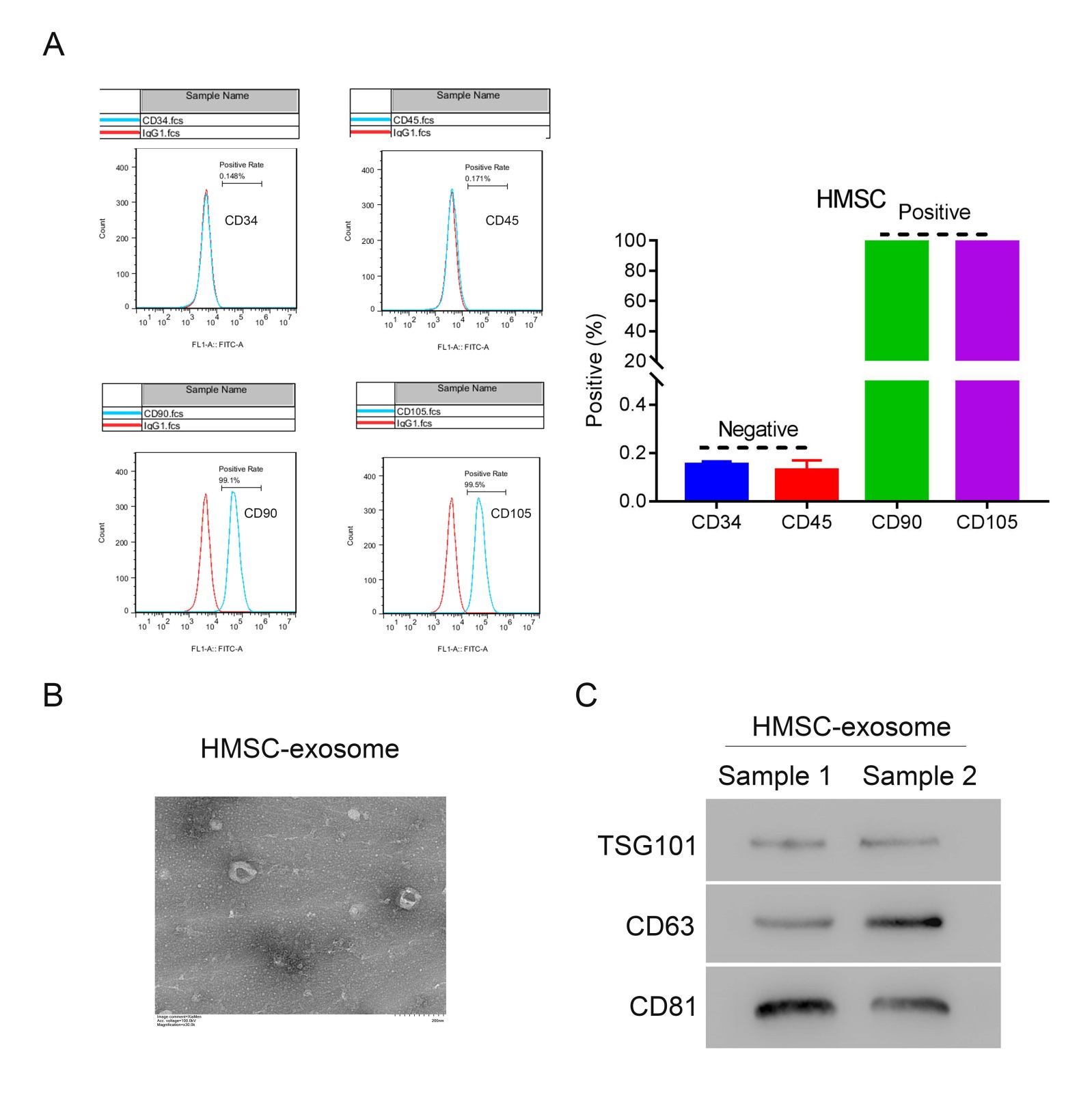
**Figure S1: Identification of** **human bone marrow mesenchymal stem cells (HMSC) and exosome.** **A.** Flow cytometer was used to examine the positive (CD90 and CD105) and negative biomarker (CD34 and CD45) for HMSC. **B.** The morphology of exosome derived from HMSC. C. Western blot was used to examine the protein levels of biomarker of exosome, including TSG101, CD63 and CD81.


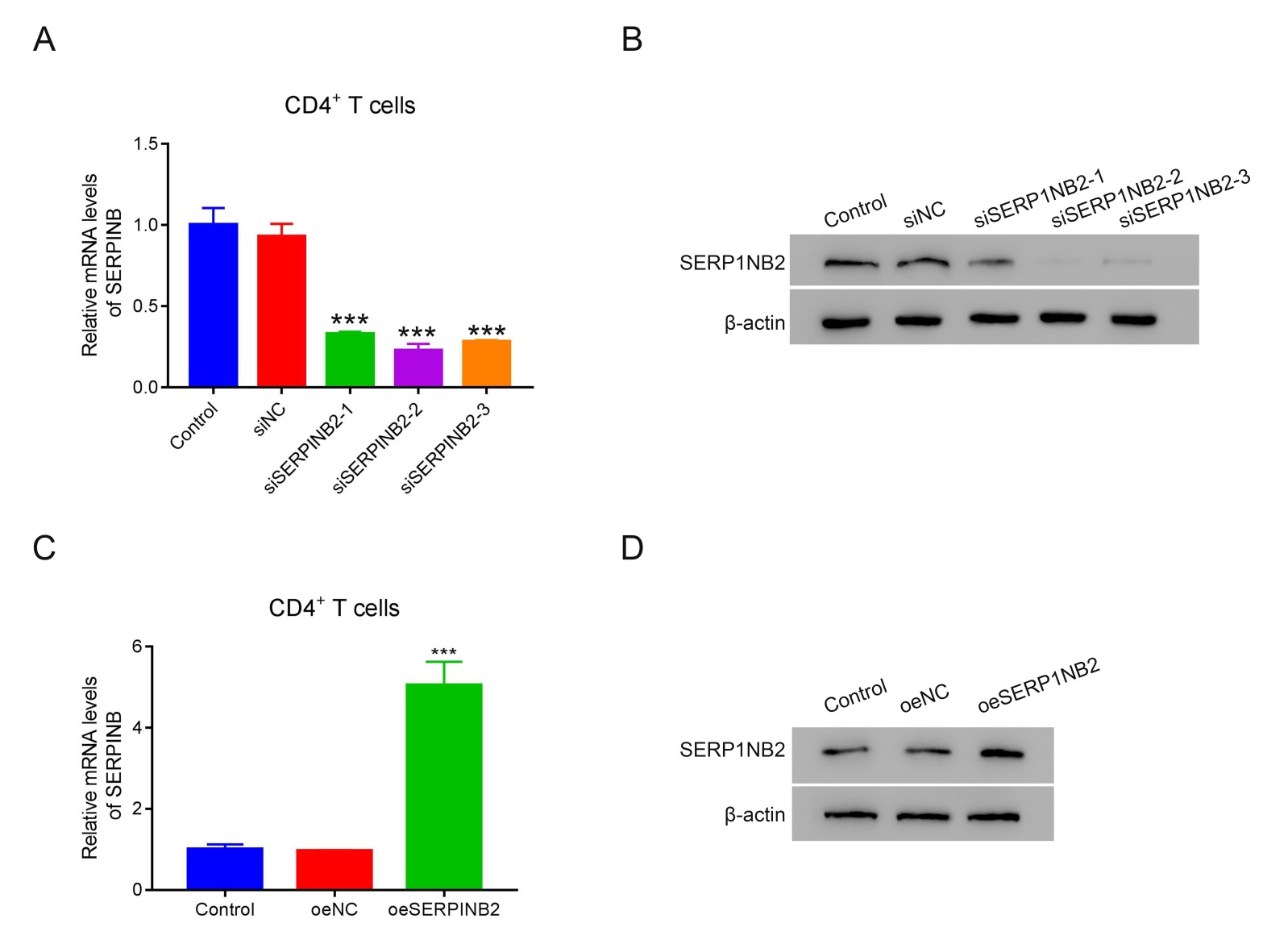


**Figure S2: Knockdown and overexpression of SERP1NB2 in normal CD4^+^ T cells. A and B.** The relative mRNA and protein levels of SERP1NB2 were deeply suppressed in CD4^+^ T cells after transfecting with siSERP1NB2-1, siSERP1NB2-2 and siSERP1NB2-3. *** p < 0.001 vs siNC. **C and D.** The relative mRNA and protein levels of SERP1NB2 were significantly overexpressed in CD4^+^ T cells after transfecting with oeSERP1NB2. *** p < 0.001 vs oeNC.
